# Supplementary material for: Land use change and ecological sensitivity in the Qingdao West Coast new area: A 30-year analysis and future scenario simulation
Source: PLoS One. 2026 Mar 5;21(3):e0339986. doi: 10.1371/journal.pone.0339986 (PMC12962472; doi:10.1371/journal.pone.0339986)
Supplement: S1 File — (DOCX) [file pone.0339986.s001.docx]

We confirm that all Figs in the manuscript were independently created by the authors using ArcGIS 10.8 software, based on publicly available geospatial data as listed in Table 1. The use of these public domain data sources (e.g., Geospatial Platform, Resource and Environment Science and Data Center, Open Street Map) is fully compliant with the journal’s Creative Commons Attribution (CC BY 4.0) license requirements and does not involve any proprietary or copyrighted base maps (such as those from Google Maps or similar commercial services).

We have uploaded the raw data, research result data, and Figs to the Figshare repository. You can access the dataset via the following link or citation: https://figshare.com/s/16f2e344582e196abbb6“
