# Supplementary material for: Land use change and ecological sensitivity in the Qingdao West Coast new area: A 30-year analysis and future scenario simulation
Source: PLoS One. 2026 Mar 5;21(3):e0339986. doi: 10.1371/journal.pone.0339986 (PMC12962472; doi:10.1371/journal.pone.0339986)
Supplement: Attachment — Submitted filename: Response to Reviewers.docx [file pone.0339986.s003.docx]

**Response to Reviewers**

**Manuscript ID:** PONE-D-25-23734

**Title:** Land Use Change and Ecological Sensitivity in the Qingdao West Coast New Area: A 30-Year Analysis and Future Scenario Simulation

**Journal:** PLOS ONE

**Dear PLOS ONE Academic Editor, and Esteemed Reviewers,**

Thank you for your letter and for the opportunity to revise our manuscript. We are grateful to you and the reviewers for the insightful and constructive comments. We believe these suggestions have significantly helped us improve the quality of our paper.

We have carefully addressed all the points raised by the Academic Editor and the two reviewers. Below, we provide a point-by-point response to the comments. We have also thoroughly revised the manuscript, which is submitted with tracked changes (‘Revised Manuscript with Track Changes’) and as a clean version (‘Manuscript’).

Furthermore, as per your request, we have added the website links for accessing each database to the submission system. We have also included these hyperlinks in the third response below.

We hope that the revised manuscript and our detailed responses will meet the high standards of **PLOS ONE**.

**Responses to the Journal Requirements**

**Point 1:** Please ensure that your manuscript meets PLOS ONE's style requirements, including those for file naming.

**Response:** We thank the editor for this reminder. We have meticulously reformatted the entire manuscript, including the title page, main body text, headings, citations, and file names, to fully comply with the **PLOS ONE** style templates.

**Point 2:** In your Methods section, please provide additional information regarding the permits you obtained for the work.

**Response:** We appreciate the need for clarity on this point. In response to this requirement, we have added a clarifying statement under the **Data Sources and Processing** section.

This study is based entirely on publicly accessible and open-source datasets obtained from the online repositories cited in Table 1. As no field work was conducted and no privately-held data were used, no specific permits were required for this research. **(lines 96-98 of the revised manuscript)**

This statement explains that the study was based entirely on public data sources and that no field work requiring specific permits was conducted.

**Point 3:** We note that Figures 1, 2, 6, 7, 8, 9, 10, 11, and 12 in your submission contain map images which may be copyrighted.

**Response:** Thank you for raising this critical point. In response to this requirement, we have added a clarifying statement under the **Supporting Information** section.

We confirm that all figures in the manuscript were independently created by the authors using ArcGIS 10.8 software, based on publicly available geospatial data as listed in **Table 1（lines 695-696 of the revised manuscript）.** The use of these public domain data sources (e.g., Geospatial Data Cloud, Resource and Environment Science and Data Center, Open Street Map) is fully compliant with the journal's Creative Commons Attribution (CC BY 4.0) license requirements and does not involve any proprietary or copyrighted base maps (such as those from Google Maps or similar commercial services). (**lines 675-680 of the revised manuscript**)

Table 1. The sources of data.

| Name | Source | Website/URL |
| --- | --- | --- |
| Base Map of the Study Area | National Platform for Common GeoSpatial Information Services | https://www.tianditu.gov.cn/ |
| Population | World Pop | https://www.worldpop.org/ |
| Elevation(DEM) | Geospatial Data Cloud | http://www.gscloud.cn |
| Slope | DEM | - |
| Aspect | DEM | - |
| Water Body Buffer | National Catalogue Service for Geographic Information | https://www.webmap.cn / |
| Road Vector Data | Open Street Map | https://openmaptiles.org/ |
| Annual Average Precipitation | Earth Resources Data Cloud | http://gis5g.com/ |
| Land Cover | Resource and Environmental Science Data Platform | https://www.resdc.cn/ |
| NDVI | National Tibetan Plateau Data Center | https://data.tpdc.ac.cn |
| Annual Average Temperature | Earth Resources Data Cloud | http://gis5g.com/ |
| Nighttime Light Data | Resource and Environmental Science Data Platform | https://www.resdc.cn/ |
| GDP | Resource and Environment Science and Data Center | https://www.resdc.cn/ |

To address your specific query, here is a breakdown of the data sources for the figures in question (To address the reviewer's valuable suggestion for a clearer methodology, we have introduced a new research flowchart as Figure 1. Please note that all subsequent figures have been renumbered to accommodate this addition.):

**Figure 2 (formerly Figure 1) - Schematic diagram of the study area: The base map, including administrative boundaries, was created using data for the "Base Map of the Study Area" from the National Platform for Common GeoSpatial Information Services (**[**https://www.tianditu.gov.cn/**](https://www.tianditu.gov.cn/)**), as detailed in Table 1.**

**Figure 3 (formerly Figure 2) - Land use changes (1990-2020): The Land Cover data used to create these maps was obtained from the Resource and Environmental Science Data Platform (**[**https://www.resdc.cn/**](https://www.resdc.cn/)**), as detailed in Table 1. Below is a screenshot of the website; for the image, we have used bilingual (Chinese and English) annotations, and key information has been highlighted in red.**

**Figure 7 (formerly Figure 6) - Single-factor ecological sensitivity maps:** These are derived maps created by us. The input data comes from various open sources as listed in Table 1.

**Elevation (DEM), Slope, and Aspect** data were obtained from the **Geospatial Data Cloud (**[**http://www.gscloud.cn**](http://www.gscloud.cn)**)**.

Below is a screenshot of the website; for the image, we have used bilingual (Chinese and English) annotations, and key information has been highlighted in red.

**Road Vector Data was obtained from Open Street Map (**[**https://openmaptiles.org/**](https://openmaptiles.org/)**).**

**Water Body** data was obtained from the **National Catalogue Service for Geographic Information (**[**https://www.webmap.cn/**](https://www.webmap.cn/)**)**.

**NDVI** data was obtained from the **National Tibetan Plateau Data Center (**[**https://data.tpdc.ac.cn**](https://data.tpdc.ac.cn)**)**.

Figures **8-13** (formerly Figures 7-12) - Comprehensive sensitivity maps and future simulations: These are the final analytical outputs of our models, generated entirely by our research team based on the synthesis of all the open-access input data.

Furthermore, we noted a recent publication in PLOS ONE, 'Zhou, J., Johnson, VC, Shi, J., Tan, ML, & Zhang, F. (2025). Multi-scenario simulation of land use change and spatio-temporal evolution of carbon storage in the Yangtze River Delta region based on the PLUS-InVEST model. PloS one, 20(1), e0316255.' This paper adopted a similar strategy for data acquisition and citation, where data were downloaded from a website and referenced accordingly, successfully leading to its publication in PLOS ONE. This suggests consistency between our approach and the journal's past practices.

In light of the above, given that all figures are our original creations, entirely based on publicly available and properly attributed data, and that we have provided detailed data source explanations and access routes, we are confident that our manuscript fully complies with the journal's Creative Commons Attribution (CC BY 4.0) license requirements

We hope this explanation satisfactorily addresses your query. We have uploaded the revised manuscript with all necessary clarifications.

**Responses to Reviewer #1**

We sincerely thank Reviewer #1 for the positive and constructive feedback**.**

**Point 1:** The novelty of the need to be clearly stated.

**Response:** We thank the reviewer for this crucial suggestion. To address this, we have substantially revised under the **Introduction** section.

While existing research has evaluated the ecological sensitivity of the West Coast New Area [17,18], our study makes three key contributions. First, we provide a comprehensive 30-year analysis (1990–2020), capturing the region's full evolution from a collection of administrative units into a national-level new area. Second, we use the advanced Patch-Generating Land Use Simulation (PLUS) model, which provides higher accuracy and better insight into land use change mechanisms than older models. Third, we introduce an integrated 'past-present-future' framework that directly links historical land use dynamics to ecological sensitivity, providing a robust scientific basis for forecasting and evaluating future scenarios. This rigorous approach offers significant value for sustainable regional development. (**lines 54-61 of the revised manuscript**)

This revised paragraph now explicitly articulates the study's novelty in terms of its temporal scale, methodological advancement, and integrated analytical framework.

**Point 2:** The study would have been better if the author/s employ the integrative approach with machine learning models. This would enhance the findings.

**Response**: This is an excellent suggestion. We agree that integrating advanced machine learning models could offer deeper insights. We have addressed this in two ways: by clarifying our current method and by incorporating your suggestion as a direction for future work.

First, we now explicitly state in the Methods section that the PLUS model we used already incorporates a Random Forest algorithm, a powerful machine learning technique, to analyze the drivers of land use change. Second, we acknowledge that a more extensive comparative analysis with other models is a valuable endeavor. Therefore, we have added this point to the new **"Limitations and Future Research Directions"** subsection.

Second, while the PLUS model's integrated Random Forest algorithm is powerful for analyz-ing driving forces, future studies could benefit from an integrative approach that compares its performance with other machine learning models, such as Artificial Neural Networks (ANN) or Support Vector Machines (SVM), to explore the complex, non-linear relationships of land use change in even greater depth. (**lines 510-514 of the revised manuscript**)

By acknowledging this limitation and proposing it as a key direction for future research, we believe the manuscript now more accurately frames the scope of its methodological contribution.

**Point 3**: Study method flowchart is missing.

**Response**: We thank the reviewer for this excellent suggestion. We agree that a flowchart is essential for visually communicating our research design and ensuring the clarity of our methods.

A new flowchart figure is inserted, visually detailing the workflow from data collection, through LUCC and ES analysis, to PLUS model simulation and final evaluation (**added as Figure 1 of the revised manuscript**).

We believe this addition directly addresses the point you raised and significantly enhances the transparency and reproducibility of our methodology.

**Point 4**: In the result section, the list of ecological sensitivity indicators and their AHP-derived weights are missing.

**Response**: Thank you for pointing this out. We have now revised under the **Ecological Sensitivity Assessment** section.

As determined by the AHP method (Table 2), Land Use Type (0.3678) and NDVI (0.2335) were the most influential factors in the ecological sensitivity assessment, highlighting the critical role of land cover and vegetation health in regional ecosystem stability. (**lines 165-167 of the revised manuscript**）

We believe this addition provides the interpretive emphasis that your comment rightly suggested was needed, directly clarifying the significance of the key factors for the reader.

**Point 5**: The Discussion section is not thorough-t typically lacks to compare the accuracy/performance of PLUS with other common models in literature, and ecological interpretation tied to sensitivity zones.

**Response**: We agree that the Discussion section needed more depth. We have significantly expanded it in two key areas:

First, to address the lack of model comparison, we have substantially revised under the **Patch-generating Land Use Simulation (PLUS) Model** section.

Land use change simulation is crucial for understanding and predicting regional development trends and for evaluating policy impacts. While previous studies have widely employed classic models like the Future Land-Use Simulation (FLUS) and Cellular Automata (CA) models [46,47], they often have limitations in capturing complex change dynamics.

To more accurately explore the underlying drivers of land use change and improve simulation accuracy, this study selected the PLUS (Patch-generating Land Use Simulation) model, a new-generation model developed by Liang Xun et al. The core innovation of the PLUS model is its coupling of two key modules: the Land Expansion Analysis Strategy (LEAS) and a CA model based on a Random Seeds and Patch Generation mechanism (CARS) [48,49].

The LEAS module, which integrates a Random Forest algorithm, offers a significant advantage by automatically uncovering the complex, non-linear relationships between land expansion and its various drivers, a capability that enhances causal inference compared to traditional regression models. Meanwhile, the CARS module excels at simulating dynamic landscape changes. By using a random seed generation and thresh-old-decreasing mechanism, this module can realistically simulate the spontaneous generation and growth of land patches based on their development probabilities [49]. This powerful capability allows the model to better explain the change mechanisms of different land use types, leading to improved simulation results and higher accuracy.

As demonstrated in multiple studies, the PLUS model provides higher simulation accuracy and a better explanation of land use change mechanisms compared to models like FLUS and CA-Markov [50], making it the ideal tool for our analysis. (**lines 189-208 of the revised manuscript**)

**Second**, to provide the requested deeper ecological interpretation, we have added a comprehensive analysis of our core finding—the "spatial polarization" of ecological sensitivity—under the **Comprehensive Ecological Sensitivity Assessment** section.

This study's core finding is the "spatial polarization of the ecological sensitivity pattern," a seemingly contradictory phenomenon where both 'non-sensitive' and 'highly sensitive' areas increased concurrently. This pattern is not a methodological artifact but rather a logical consequence of China's unique spatial planning paradigm, where 'development' and 'conservation' are spatially decoupled. It is driven by two powerful, parallel, and spatially explicit forces:

Driver One: The National-Level New Area Development Strategy, Concentrated in Coastal Plains.

This policy-driven, high-intensity urbanization process is spatially concentrated in the northeastern, central, and southeastern coastal zones, which are characterized by flat terrain, high concentrations of economic activity, and the most intensive urban construction—namely, the three core economic functional clusters of Huangdao, Jiaonan, and Dongjiakou. The rapid urban and industrial expansion inevitably requires vast amounts of land, primarily sourced from the encroachment upon surrounding agricultural lands and transitional zones of moderate ecological sensitivity. This directly leads to a significant expansion of 'non-sensitive' areas, which represent high levels of anthropogenic disturbance, within these specific zones.

Driver Two: The Mandatory Ecological Conservation Policy, Focused on Inland Mountainous Regions.

Concurrently, mandatory conservation policies, epitomized by the "ecological protection red line," have precisely targeted the region's most critical ecological function areas: the three core inland mountain systems of Xiaozhu Mountain, Dazhu Mountain, and Cangma Mountain-Tiejue Mountain. These policies have provided strict legal protection for these areas and have promoted a series of ecological restoration projects, such as afforestation and mountain habitat rehabilitation. According to this study's evaluation framework, high vegetation cover and forest land are the core factors assigned the highest weights for 'high sensitivity'; therefore, these successful ecological restoration efforts are accurately identified by our model as a significant increase in the extent of 'highly sensitive' and 'extremely highly sensitive' zones.

Therefore, this 'polarization' pattern authentically reflects the unique spatial reality of China's rapid development, where 'high-intensity construction' and 'mandatory conservation' operate as parallel strategies in functionally zoned spaces. The direct trade-off is the rapid transformation and squeezing of the vast 'moderately sensitive areas' that act as intermediate buffer zones, ultimately resulting in a landscape where development and conservation zones are highly differentiated and spatially explicit. This serves as strong evidence for the effectiveness of our assessment framework, as it successfully captures this complex and policy-driven regional evolution. **(lines 383-412 of the revised manuscript)**

Finally, based on this deeper interpretation, we have added a new section to the manuscript titled "**Policy Implications**" to provide actionable recommendations.

Based on these findings, we propose three specific policy recommendations for the QWCNA and similar coastal new areas. First, planning authorities should prioritize the establishment of 'green corridors' connecting the high-sensitivity zones of Xiaozhu, Dazhu, and Cangma-Tiejue mountains to enhance habitat connectivity and combat fragmentation. Second, the rapidly shrinking moderately sensitive zones, primarily agricultural land, should be designated as 'ecological buffer zones' with stricter development controls to prevent further urban encroachment. Third, future urban expansion should be directed exclusively towards infill development within the existing non-sensitive and low-sensitivity areas, promoting a more compact and efficient urban form consistent with the EP scenario's optimal land use structure. (**lines 494-502 of the revised manuscript**)

We believe these extensive revisions—providing a clear justification for our model choice, an in-depth ecological interpretation of our core findings, and actionable policy recommendations—fully address your concerns and significantly enhance the depth and impact of our manuscript.

**Point 6**: The number of references cited for this work are only 37. More recent and localized studies need to be included.

**Response**: We agree completely. We have conducted a thorough literature review and have added over 22 new references. These include more recent publications and studies specifically focused on the Shandong Peninsula, Qingdao, and other comparable coastal new areas in China. This has enriched our **Introduction and Discussion** sections, providing a stronger contextual and comparative foundation for our work.

**Responses to Reviewer #2**

We sincerely thank Reviewer #2 for the rigorous and challenging critique, which has pushed us to substantially strengthen the methodological foundations of our manuscript.

**Point 1**: The manuscript addresses an important topic, but several methodological issues significantly limit its scientific rigor. The ecological sensitivity assessment framework appears to lack adequate theoretical foundation and validation. The selection of seven factors and their weighting through AHP relies heavily on subjective expert judgment (only 5 experts), introducing substantial bias into what should be an objective ecological assessment. The grading criteria in Table 4 seem arbitrary—for example, the assignment of "North" aspect as having the highest sensitivity lacks clear ecological justification. More critically, the study conflates ecological sensitivity with concepts like naturalness and conservation value without providing clear definitions or demonstrating how the chosen factors actually measure ecosystem sensitivity to disturbance. The authors should either validate their sensitivity framework against independent ecological data or adopt established ecological vulnerability assessment methods from the literature.

**Response**: We are grateful for this fundamental and detailed critique. We have addressed each of your key points through several major revisions.

First, to address the concerns about the theoretical foundation and the conflation of key concepts, we have made several clarifications:

We have revised the **Introduction** to provide a clear and standard definition of ecological sensitivity, grounding it in the literature.

Ecological sensitivity refers to the degree of an ecosystem's response to natural environmental changes and human disturbances within a region, reflecting the likelihood of ecological problems arising when the system is impacted by these factors [4-6]. Essentially, ecological sensitivity assessment is a method for identifying potential ecological problems under existing natural and anthropogenic backgrounds, providing valuable information for their prevention and management [7-9]. The evaluation of ecological sensitivity is of great significance for regional environmental protection and the rational development of natural resources [10-12]. It can guide practices such as ecological zoning, landscape planning, and urban planning, thereby laying a solid foundation for regional ecological civilization construction and the harmonious development between humanity and nature [13-16]. (**lines 45-53 of the revised manuscrip**t)

The original manuscript's linkage of sensitivity to "naturalness" was indeed an imprecise expression that has now been corrected. We have also clarified in the Discussion that the spatial overlap between high-sensitivity zones and designated conservation areas is a key finding reflecting policy impacts, rather than an issue of conceptual conflation

Land cover type directly indicates the complexity and stability of the ecosystem. Forest ecosystems, with their complex structure, high biodiversity, and critical roles in soil and water conservation, are highly susceptible to irreversible damage from disturbances, thus warranting the highest sensitivity rating. This is followed by grasslands and water bodies. Conversely, built-up land, being an already highly disturbed and artificial system, is deemed non-sensitive to further ecological disruption. **(lines177-182 of the revised manuscript)**

High Sensitivity Areas (High and Extremely High Sensitivity Areas): These cluster mainly around mountain ranges such as Xiaozhu Mountain, Cangma-Tiejue Mountain, and Dazhu Mountain. These areas typically have higher elevations, complex terrain, and ecosystems that are relatively pristine or fragile, thus exhibiting weak resistance to external disturbances. This results in their high ecological sensitivity. It is a key finding that these highly sensitive zones show a strong spatial correlation with designated land uses such as ecological protection zones, water source conservation areas, and important tourism resource sites, including numerous national parks and scenic spots. Human activity is relatively limited in these zones, which contributes to the preservation of their sensitive ecological characteristics. **(lines 363-370 of the revised manuscript)**

Second, to address your critical point about the grading criteria seeming arbitrary, we have added detailed ecological justifications. We agree that this was needed, and our response is not to assert that our criteria are absolute, but to show they are based on established scientific principles adapted to our region. For the "North" aspect you highlighted:

Variation in slope aspect causes differences in the duration and intensity of solar radiation. In the study area, north-facing slopes are shady slopes, while south-facing slopes are sunny slopes, which receive relatively greater duration and amounts of solar radiation. They are followed, in descending order, by southeast and southwest-facing slopes, then east and west-facing slopes, with northeast and northwest-facing slopes receiving the least. Therefore, sunny slopes, with a more favorable microclimate, tend to support healthier ecosystems with stronger resilience to environmental stress, and are thus classified as having lower sensitivity. Conversely, shady slopes, with a less favorable microclimate, exhibit higher ecological sensitivity.

North-facing slopes received the highest sensitivity rating because of distinct microclimatic conditions, such as reduced solar radiation and potentially different moisture availability. (**lines 176-177 of the revised manuscript)**

Third, regarding the subjectivity of the AHP weighting process, we have clarified our robust approach:

While we acknowledge the inherent subjectivity in any expert-led process, our AHP was structured through a Delphi Method to build consensus among experts from diverse fields (ecology, geography, urban planning), thereby mitigating individual bias. Critically, our final weight allocation passed an objective consistency test (CR = 0.0081, see Table 3), which mathematically validates the logic and reliability of our expert judgments. Furthermore, a comparison with similar studies confirms that our weight assignments are well-aligned with established findings in the field.

Finally, we agree on the value of exploring different weighting methodologies and validating the framework against independent data. We have acknowledged these important points in the new "**Limitations and Future Research Directions**" section.

First, the determination of factor weights in the ecological sensitivity assessment, while structured through an AHP-Delphi process, contains a degree of subjectivity. Future studies could employ more objective weighting methods or conduct a sensitivity analysis to test how variations in weights affect the final sensitivity patterns. (**lines 506-509 of the revised manuscript**)

We also agree that validating the framework against independent ecological data, as you suggested, would be a valuable next step in this line of research.

**Point 2**: The study lacks essential uncertainty analysis and sensitivity testing that would demonstrate the robustness of its findings. The PLUS model validation, while showing acceptable accuracy (Kappa = 0.782), is limited to a single time period and may not represent model performance under different conditions. The three development scenarios lack detailed quantitative constraints and realistic policy implementation details. Additionally, the simultaneous increase in both non-sensitive areas (due to urbanization) and extremely high sensitivity areas requires better explanation—this counterintuitive pattern suggests potential issues with the sensitivity classification system. The authors should incorporate uncertainty bounds for all major results, conduct sensitivity analysis on key methodological choices, and provide more rigorous validation of both the ecological sensitivity framework and the scenario modeling approach. Statistical significance testing for observed temporal changes would also strengthen the analysis considerably.

**Response**: We are sincerely grateful for this rigorous and comprehensive critique, which has prompted us to substantially strengthen our manuscript's analytical depth and robustness. We have addressed each of your key points through several major revisions.

First, regarding the model validation and scenario rigor, we have clarified our approach to ensure its scientific basis is transparent.

On Model Validation: Our approach—simulating the most recent known time point (2020) based on the preceding historical period—is a widely established practice in land use change modeling. It provides a robust test of the model's parameterization for our specific study area. The validation results, showing a high Kappa coefficient of 0.782 and an overall accuracy of 0.886, provide strong confidence in the model's suitability for near-term future simulation.

On Scenario Constraints: Our scenarios are not arbitrary. They are systematically constructed with the Natural Development (ND) scenario as a baseline. The Urban Development (UD) and Ecological Protection (EP) scenarios were then derived by applying quantitative modifications to the transition matrix based on two primary sources: 1) Official Planning Documents, such as the "Qingdao West Coast New Area Territorial Spatial Zoning Plan (2021-2035)," and 2) Relevant Academic Literature. The specific parameter adjustments are detailed in the "**Setting Up Future Multi-Scenario Modes**" subsection of the Methods. **(lines 214-242 of the revised manuscript)**

Second, to provide the requested deeper explanation for the "counterintuitive pattern" of concurrent increases in non-sensitive and highly sensitive areas, we have added a comprehensive analysis to the **Comprehensive Ecological Sensitivity Assessment** section. We clarify that this is not a methodological flaw but a core finding we term the "spatial polarization of ecological sensitivity".

This pattern is not a methodological artifact but rather a logical consequence of China's unique spatial planning paradigm, where 'development' and 'conservation' are spatially decoupled. It is driven by two powerful, parallel, and spatially explicit forces:

Driver One: The National-Level New Area Development Strategy, Concentrated in Coastal Plains. This policy-driven, high-intensity urbanization process is spatially concentrated in the northeastern, central, and southeastern coastal zones...

Driver Two: The Mandatory Ecological Conservation Policy, Focused on Inland Mountainous Regions. Concurrently, mandatory conservation policies, epitomized by the "ecological protection red line," have precisely targeted the region's most critical ecological function areas: the three core inland mountain systems...

Therefore, this 'polarization' pattern authentically reflects the unique spatial reality of China's rapid development... This serves as strong evidence for the effectiveness of our assessment framework, as it successfully captures this complex and policy-driven regional evolution. **(lines 383-411 of the revised manuscript)**

Third, flowing from this deeper interpretation, we have added a new section to the manuscript titled "**Policy Implications**" to provide actionable recommendations based on our findings

Based on these findings, we propose three specific policy recommendations for the QWCNA and similar coastal new areas. First, planning authorities should prioritize the establishment of 'green corridors' ... Second, the rapidly shrinking moderately sensitive zones, primarily agricultural land, should be designated as 'ecological buffer zones' ... Third, future urban expansion should be directed exclusively towards infill development ... **(lines 494-502 of the revised manuscript)**

Finally, to address the reviewer's crucial points on uncertainty, sensitivity analysis, and statistical testing, we have added a new section to the manuscript titled "**Limitations and Future Research Directions**."

We acknowledge the significant value of these in-depth analyses. However, given the established scope of the current study and the journal's length guidelines, we have incorporated them as priorities for future work rather than expanding the current manuscript. The new section now explicitly details these points to ensure transparency and guide future research.

Third, while our PLUS model validation showed high accuracy for the historical period, all future simulations are subject to uncertainty. The scenarios are based on current policies and trends, which may shift over time. Future work could incorporate dynamic, adaptive scenarios that respond to potential policy or climate changes. **(lines 515-518 of the revised manuscript)**

Fourth, our analysis of temporal change is based on the net area changes of sensitivity classes. While substantial, this does not include pixel-level statistical significance testing of the changes, which could provide finer-grained insights. **(lines 519-521 of the revised manuscript)**

This addition ensures that we acknowledge these important considerations and outline a clear path for future research, directly addressing the reviewer's insightful critique.

We are confident that these extensive revisions have substantially improved the manuscript. We thank you again for your time and consideration.

Sincerely,

Dr. Tong Zhou

(Corresponding Author)

College of Civil Engineering and Architecture,

Shandong University of Science and Technology, Qingdao, China.

Email: zhoutong@sdust.edu.cn
